# Soluble Interleukin-15 Complexes Are Generated *In Vivo* by Type I Interferon Dependent and Independent Pathways

**DOI:** 10.1371/journal.pone.0120274

**Published:** 2015-03-10

**Authors:** Scott M. Anthony, Megan E. Howard, Yared Hailemichael, Willem W. Overwijk, Kimberly S. Schluns

**Affiliations:** 1 Departments of Immunology, The University of Texas MD Anderson Cancer Center, Houston, Texas, United States of America; 2 Immunology Graduate Program, The University of Texas Graduate School of Biomedical Sciences at Houston, Houston, Texas, United States of America; 3 Melanoma Medical Oncology, The University of Texas MD Anderson Cancer Center, Houston, Texas, United States of America; University of Iowa, UNITED STATES

## Abstract

Interleukin (IL)-15 associates with IL-15Rα on the cell surface where it can be cleaved into soluble cytokine/receptor complexes that have the potential to stimulate CD8 T cells and NK cells. Unfortunately, little is known about the in vivo production of soluble IL-15Rα/IL-15 complexes (sIL-15 complexes), particularly regarding the circumstances that induce them and the mechanisms responsible. The main objective of this study was to elucidate the signals leading to the generation of sIL-15 complexes. In this study, we show that sIL-15 complexes are increased in the serum of mice in response to Interferon (IFN)-α. In bone marrow derived dendritic cells (BMDC), IFN-α increased the activity of ADAM17, a metalloproteinase implicated in cleaving IL-15 complexes from the cell surface. Moreover, knocking out ADAM17 in BMDCs prevented the ability of IFN-α to induce sIL-15 complexes demonstrating ADAM17 as a critical protease mediating cleavage of IL-15 complexes in response to type I IFNs. Type I IFN signaling was required for generating sIL-15 complexes as in vivo induction of sIL-15 complexes by Poly I:C stimulation or total body irradiation (TBI) was impaired in IFNAR-/- mice. Interestingly, serum sIL-15 complexes were also induced in mice infected with Vesicular stomatitis virus (VSV) or mice treated with agonistic CD40 antibodies; however, sIL-15 complexes were still induced in IFNAR-/- mice after VSV infection or CD40 stimulation indicating pathways other than type I IFNs induce sIL-15 complexes. Overall, this study has shown that type I IFNs, VSV infection, and CD40 stimulation induce sIL-15 complexes suggesting the generation of sIL-15 complexes is a common event associated with immune activation. These findings reveal an unrealized mechanism for enhanced immune responses occurring during infection, vaccination, inflammation, and autoimmunity.

## Introduction

Interleukin (IL)-15 is a tightly regulated cytokine that mediates the development and long-term homeostasis of memory CD8 T cells, Natural Killer (NK) cells, invariant NKT cells, TCRγδ+ T cells and intraepithelial lymphocytes [1,2]. Multiple mechanisms exist that restrict the transcriptional and translational expression of IL-15 [3–5]. In addition, because IL-15 is co-expressed with the high affinity IL-15Rα chain, IL-15 becomes associated with IL-15Rα in the endoplasmic reticulum and is subsequently shuttled to the cell surface as a complex [6]. This cell surface complex of IL-15 and IL-15Rα is capable of stimulating neighboring cells via the IL-2/15Rβ and γC complex during a cell-cell interaction via a mechanism called transpresentation [6]. Transpresentation is believed to be the major mechanism mediating IL-15 functions during homeostasis, delivering a low and constitutive IL-15 signal to IL-15 responsive cells [7–9]. Nonetheless, surface IL-15Rα/IL-15 can also be cleaved to form soluble IL-15Rα/IL-15 complexes (sIL-15 complexes)[7,10]. *In vitro* and *in vivo* production of sIL-15 complexes have been observed after stimulation with Toll-like receptor (TLR)3 and TLR4 agonists, Poly I:C and Lipopolysaccharide (LPS), respectively [7]. Additionally, sIL-15 complexes are transiently increased in sera of mice and human patients undergoing lymphodepletion induced by total body irradiation (TBI) or chemotherapy [10]. Since sIL-15 complexes have agonist properties and exhibit an approximate 50–100 fold greater proliferative effect over recombinant IL-15 alone on responding CD8 T cells [11,12], the generation of sIL-15 complexes is likely an important, yet undelineated mechanism regulating IL-15 responses. Unfortunately, few studies have examined regulatory mechanisms generating sIL-15 complexes.

Many types of immune activation and inflammatory responses are associated with increases in IL-15 and IL-15Rα expression and enhanced IL-15 responses. For example, IL-15 expression is increased in autoimmune diseases, including Rheumatoid Arthritis, Psoriasis, and inflammatory bowel diseases, where it is thought to contribute to immune cell activation [13–15]. In addition, transcription of IL-15 and IL-15Rα is increased during numerous types of viral and bacterial infections [16–18]. Regulation of IL-15 through stimulation of specific immune pathways is evident as TLR ligands, Type I IFNs and agonistic anti-CD40 antibody increase IL-15 expression as well as cell surface IL-15 expression [7,19–24]. Moreover, enhanced IL-15 responses have also been reported upon stimulation with Poly I:C, IFN-α, or anti-CD40 Ab [7,24–26]. While elevated IL-15 and IL-15Rα expression are clearly associated with immune activation and inflammation, whether sIL-15 complexes are generated during these situations is not clear. Since Bergameschi et al [10] found that all serum IL-15 present was associated with sIL-15Rα, past studies reporting elevated IL-15 may not have accurately distinguished IL-15 from sIL-15 complexes or missed sIL-15 detection altogether. Hence, the overall aim of this study was to identify immune stimuli that induce sIL-15 complexes and determine the importance of type I IFNs; these efforts lead to the discovery that numerous types of immune stimulation upregulate sIL-15 complexes and do so using both IFN-dependent and independent pathways. These studies provide a new understanding of mechanisms regulating IL-15 responses during non-steady state conditions; albeit by sIL-15 complexes, through transpresentation, or the combination.

## Materials and Methods

### Animal experiments and ethic statement

C57Bl/6 mice were purchased from NCI/Charles River. ADAM17 ^fl/fl^ [27] mice were purchased from Jackson Laboratories (Bar Harbor, MN). IFNAR1-/- mice [28] were provided by Dr. Paul W. Dempsey (Department Of Microbiology and Molecular Genetics, University of California, Los Angeles and Dr. Tadatsugu Taniguchi, Department of Immunology, Tokyo University, Japan) to W. Overwijk and crossed to the C57Bl/6 background. All mice were maintained under specific pathogen-free conditions at the institutional animal facility. The animal facility is fully accredited by the Association of Assessment and Accreditation of Laboratory Animal Care International. All animal procedures were conducted on mice between 8–12 weeks of age, in accordance with the animal care and use protocols (100409934 and 070807332) approved by the Institutional Animal Care and Use Committee at the UT MD Anderson Cancer Center. Retro-orbital blood was obtained from mice anaesthetized by inhalation with a mixture of 2% isoflurane/98% oxygen. For all manipulations, animals were monitored and efforts were made to minimize suffering. At the designated times, the animals were sacrificed according to institutional guidelines and blood and tissues were collected for analyses.

Mice were injected with pORF-IFNα 5 (2 μg) (InvivoGen, SanDiego, CA) to induce *in vivo* IFN-α production or empty vector plasmid pORF (InvivoGen) in 2mL saline via hydrodynamic injection as previously described [24]. For VSV infection, mice were infected i.v with 1 x 10^6^ PFU (VSV, Indiana strain). For Poly I:C stimulation, mice were administered Poly I:C i.p (150 μg, Sigma, St. Louis, MO). For TBI, mice were exposed to a cesium irradiation source at the indicated doses. CD40 stimulation was carried out by injecting i.p. anti-CD40 monoclonal Ab (clone FGK 4.5, at the indicated doses BioXcell, Upper Heyford, UK). Peripheral blood was collected from sacrificed mice via cardiac puncture and on some occasions from the retro-orbital cavity prior to treatment from the same mice. Blood was allowed to clot and centrifuged to separate serum.

### Analysis of Cytokine Expression

ELISAs specific for murine soluble IL-15Rα/IL-15 complexes (eBioscience, San Diego, CA) and murine IFN-α (detects all 14 IFN-α subtypes, PBL Biomedical Laboratories) were performed according to manufacturer’s recommendations. Cell surface IL-15 was detected in splenic myeloid cells isolated directly *ex vivo* as previously described [29]. Briefly, cell surface IL-15 was detected with polyclonal rabbit anti-IL-15-biotin (Peprotech, Rocky Hill, NJ) followed by streptavidin-APC (Jackson ImmunoResearch). Background staining was determined by staining analogous populations with a biotinylated Ig control (Jackson ImmunoResearch). The following monoclonal (m) Abs were purchased from BD Biosciences (San Jose, CA), eBiosciences, or BioLegend: CD19, CD3, DX5, CD11b, CD11c. Expression of CD19, CD3 and DX5 was used to define lineage+ cells. Flow cytometric data were acquired with a LSRII (BD Biosciences) or LSRFortessa (BD Biosciences) and analyzed with Flowjo software version 9.7.6 (Flowjo LLC, Ashland, OR).

### Bone marrow derived dendritic cells (BMDCs)

BMDCs were generated using GM-CSF stimulation as previously described [30]. Briefly, BM cells were flushed from femurs of indicated mice, dissociated, and treated with Tris ammonium chloride to lyse red blood cells. BM cells were then cultured in RPMI Complete Medium (CM) at a concentration of 1x10^6^ cells/mL. CM RPMI contains 2.5,mM HEPES, 5.5x10^-5^ M 2-mercaptoethanol, 100U/ml penicillin, 100 μg/ml streptomycin, 5mM glutamine and 10% fetal calf serum supplemented with 10 ng/ml GM-CSF (R&D Systems, Minneapolis, MN) at 37°C with 5% CO_2._ BM cells were passaged 1:2, 3–4 days later with fresh CM containing GM-CSF (10ng/mL). After 6 days, cells were given fresh media and cultured in the absence or presence of Poly I:C (50 μg/mL, Sigma) or IFNα (300U/mL, PBL Laboratories, Piscataway,NJ). Supernatants were collected and analyzed for sIL-15 complexes using ELISA. For analysis of ADAM17 expression, CD11c+CD11b+ BMDCs were stained with ADAM17 Ab (purified polyclonal Rabbit IgG, eBioscience) or control Rabbit IgG (JacksonImmunoResearch), followed by Donkey anti-Rabbit APC (JacksonImmunoResearch).

For Tat-Cre *in vitro* recombination, BM cells were collected from the indicated mice, washed twice and cultured in serum-free media with TAT-Cre for 1 hour at 37°C with 5% CO_2_ (10 μg per 1X10^6^ cells, Protein and Proteomics Core Facility, The Children's Hospital of Philadelphia). Transduced BM cells were then washed twice with serum free media followed by culturing in CM containing GM-CSF for 6 days to generate BMDC. After 6 days, BMDC were stimulated as described and culture supernatants were collected for analysis of sIL-15 complexes.

### Statistical Analysis

Statistical differences were determined by a two-tailed Students *t* test. * indicates p<0.05. Analyses were performed using GraphPad Prism, version 6 (GraphPad Software, San Diego, CA) or Microsoft Excel 2010(Redmond, WA)

## Results

### IFN-α induces the generation of sIL-15 complexes

**Fig 1:**
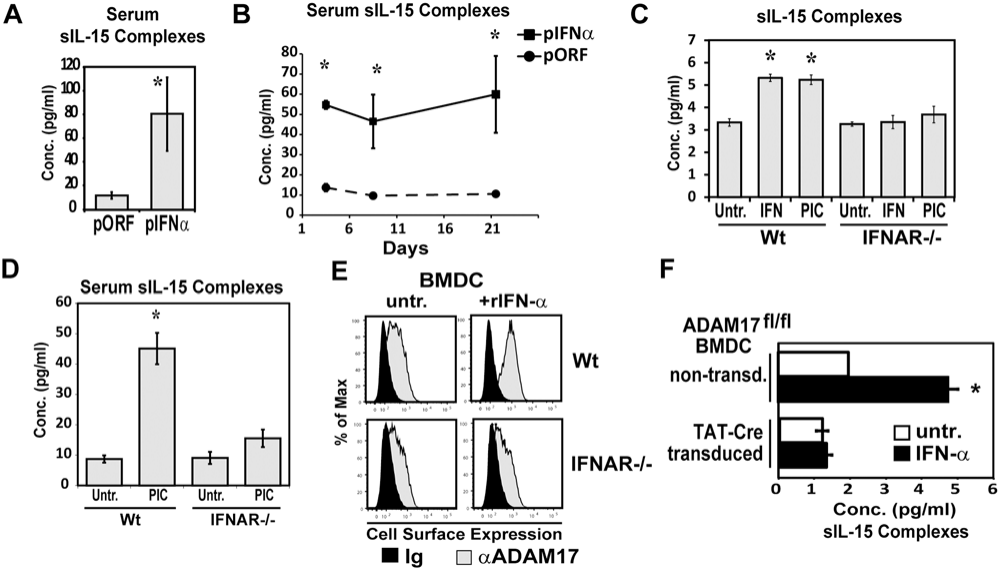
IFN-α increases sIL-15 complexes *in vitro* and *in vivo*. (A) A plasmid encoding IFN-α, (pIFNα) or an empty vector control plasmid (pORF) were injected i.v. by hydrodynamic injection. Serum was isolated A) 48 hrs post injection and B) after various times post plasmid injection. Levels of sIL-15 complexes in serum were measured using ELISA. n = 2–3 mice/group. Data is representative of three experiments. C) Culture supernatants were collected from WT and IFNAR1-/- BMDC treated with rIFN-α (300 U/mL) or Poly I:C (50 μg/mL) for 24 hr. Levels of sIL-15 complexes in culture supernatants were measured using ELISA. Data is representative of three experiments. D) Levels of sIL-15 complexes in serum from Wt and IFNAR1-/- mice 24 hours after treatment with Poly I:C (150 μg, i.p.) were measured using ELISA. n = 3 mice/group. Data is representative of three experiments. E) Cell-surface ADAM17 expression in WT and IFNAR1-/- BMDCs was detected by indirect immunofluorescence staining and flow cytometric analysis after either no treatment or stimulation with rIFN-α (300U/mL) for 24 hr. F) Non-transduced and TAT-Cre transduced BMDCs generated from ADAM17^fl/fl^ mice were left untreated (open bars) or stimulated with rIFN-α (300U/mL) (filled bars). Culture supernatants were collected 24hrs later and analyzed for sIL-15 complexes. n = 1–3 wells/group. Data is representative of two experiments, * indicates p<0.05.

Previous studies have shown that Type I IFNs increase IL-15 mRNA and protein expression [18–20]. Moreover, our past studies showed that IFN-α increases surface IL-15 expression on DCs [24,31]; however, whether there are concomitant effects on the production of sIL-15 complexes has not been determined. As such, we investigated if Type I IFNs affect the generation of sIL-15 complexes. To induce IFN-α production *in situ*, mice were given plasmid encoding IFN-α (pORF-IFN-α) or control plasmid (pORF) via hydrodynamic injections. In mice given pORF-IFN-α, sIL-15 complexes were significantly increased in the serum 48h post injection (Fig. 1A). This procedure promotes long term production of IFN-α *in vivo* [24]. For as much as 21 days after injection of pORF-IFN-α, levels of sIL-15 complexes remained high (Fig. 1B) demonstrating that continual Type I IFN-signaling *in vivo* is sufficient for sustaining production of sIL-15 complexes. To determine if IFN-α acts directly on DCs, BMDC generated from WT and IFNAR-/- mice were treated with rIFN-α (300U/mL) or Poly I:C (50μg/mL). Both Poly I:C and rIFN-α increased the levels of sIL-15 complexes in supernatants from WT BMDCs but not IFNAR-/- DCs showing that rIFN-α directly induces production of sIL-15 complexes (Fig. 1C). To determine the requirement for Type I IFNR signaling *in vivo*, IFNAR-/- mice were analyzed after treatment with Poly I:C, which induces IFN-α as well as sIL-15 complexes [7]. sIL-15 complexes were not efficiently induced by Poly I:C in IFNAR-/- mice (Fig. 1D) indicating that Type I IFNR signaling is important for production of sIL-15 complexes in response to TLR3 stimulation.

Since A Disintegrin And Metalloprotease (ADAM) 17 can cleave IL-15Rα from the surface of cells *in vitro* [32,33], ADAM17 is currently suspected to cleave IL-15Rα /IL-15 complexes into a soluble form. Since translocation of ADAM17 to the cell surface is indicative of ADAM17 activity [34], cell surface ADAM17 expression by BMDCs was measured using flow cytometry as a means to assess the ability of Type I IFNs to regulate ADAM17 activity. Stimulation with IFNα for 24 hrs strongly up-regulated the cell-surface expression of ADAM17 in WT BMDCs but exhibited no effect on cell-surface ADAM17 expression in IFNAR-/- BMDCs (Fig. 1E). To delete ADAM17 in BMDC, BMDC were generated from ADAM17^flox/flox^ (ADAM17^fl/fl^) mice in the absence or presence of TAT-Cre to mediate *in vitro* recombination and deletion of ADAM17. While IFN-α increased sIL-15 complexes in non-transduced BMDCs, there was a complete lack of IFNα-induced sIL-15 complexes in ADAM17-deficient BMDCs (Fig. 1F) showing induction of sIL-15 complexes in BMDC by IFN-α is dependent on ADAM17. Overall, these data provide evidence that type I IFNs induce cleavage of IL-15 complexes.

### Increases in sIL-15 complexes after TBI are dependent on IFNAR signaling

**Fig 2:**
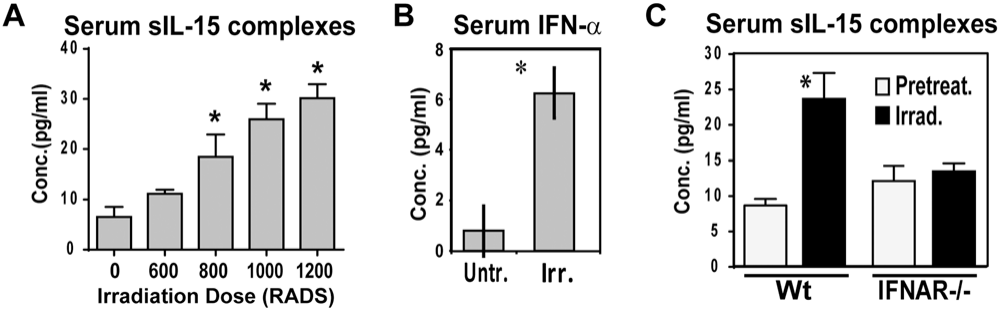
Type I IFN signaling is required for increasing sIL-15 complexes after total body irradiation. Serum was isolated 24 hours after treating WT mice with varying levels of total body irradiation. Levels of sIL-15 complexes in serum were measured using ELISA. n = 3–4 mice/group. Induction of sIL-15 complexes after TBI has been observed in at least 8 experiments. (B) IFN-α levels were measured in serum isolated from WT mice 24hours after treatment with 1000 RADs of total body irradiation. One of two experiments is shown. (C) Serum was isolated from WT and IFNAR1-/- mice 24 hours after treatment with TBI (1000 RADs). Levels of sIL-15 complexes in serum were measured using ELISA. n = 3–4 mice/group; data is representative of 4 experiments. * indicates p<0.05.

Since TBI has been shown to increase serum sIL-15 complexes and previous reports have shown Type I IFNs mRNA increases after local irradiation [10,35], we asked if Type I IFNs proteins were important for induction of sIL-15 complexes after TBI. Similar to previous reports [10], TBI increased the levels of sIL-15 complexes in serum 24h post treatment and did so in a dose dependent manner (Fig. 2A). Additionally, systemic IFN-α protein levels were increased in mice given TBI (Fig. 2B). Using IFN-β specific ELISA, IFN-β was not detected in any of the samples (data not shown). To assess the importance of IFN-α in inducing sIL-15 complexes after TBI, IFNAR-/- mice were analyzed. In the absence of IFNAR, sIL-15 complexes were not induced to a significant level after TBI (Fig. 2C) suggesting Type I IFNs are an important signal inducing sIL-15 complexes in response to TBI.

### Serum sIL-15 complexes are increased after VSV infection

**Fig 3:**
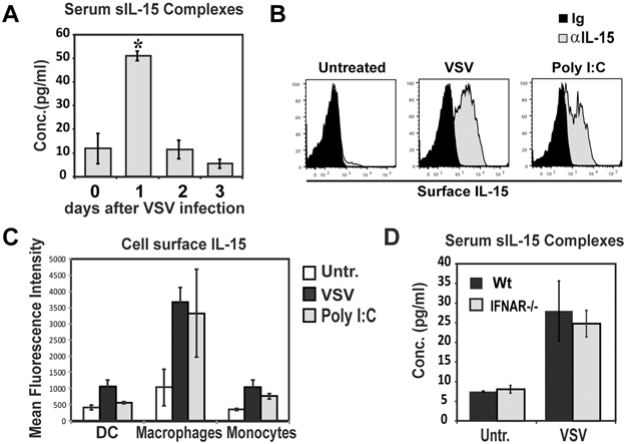
Viral infection transiently induces sIL-15 complexes *in vivo* independent of IFN signaling. A) Serum levels of sIL-15 complexes in Wt mice, at indicated times post VSV infection (1x10^6^ PFU/ mouse, i.v.) (n = 2–5 mice/group), one representative experiment of five total is shown. B,C) Cell surface IL-15 expression in myeloid cells one day after VSV infection or Poly I:C injection (150 μg/mouse, i.p.) as determined by immunofluorescence staining and flow cytometry. Histograms depict representative staining with control Ig (black histogram) and anti-IL-15 Ab (grey histograms) in macrophages (lineage-CD11b+CD11c+F480+). Graph shows average MFI of IL-15 expression in DCs (lineage-CD11b+/-CD11c+F480), macrophages, and monocyte (lineage-CD11b+CD11c-F480-) of indicated mice. n = 3 mice/group, one representative of three experiments shown. D) Levels of sIL-15 complexes in serum from WT and IFNAR-/- mice one day post VSV infection. n = 3–4 mice/group; data is representative of three experiments. * indicates p<0.05.

Vesicular Stomatitis Virus (VSV) infection induces high levels of Type I IFNs as well as increases IL-15 expression in IL-15 reporter mice [18]. Therefore, we asked if VSV infection induces the generation of sIL-15 complexes. One day after VSV infection, levels of sIL-15 complexes in serum were transiently increased, returning to baseline two days post infection (Fig. 3A). Since IFN-α increases sIL-15 complexes as well as cell surface IL-15 [24], we examined how VSV affects cell surface IL-15 expression. Interestingly, cell surface IL-15 by splenic DCs, macrophages, and monocytes was significantly increased one day after VSV infection (Fig. 3B, C) showing that similar to IFN-α stimulation, increases in cell surface IL-15 coincide with appearance of sIL-15 complexes. Unexpectedly, VSV efficiently induced sIL-15 complexes in IFNAR-/- mice (Fig. 3D) indicating other pathways are involved in VSV-mediated induction of sIL-15 complexes. Overall, these data show for the first time that sIL-15 complexes are transiently increased early in a VSV infection in a manner independent of Type I IFN signaling.

### sIL-15 complexes are transiently increased by CD40 stimulation independent of type I IFN signaling

**Fig 4:**
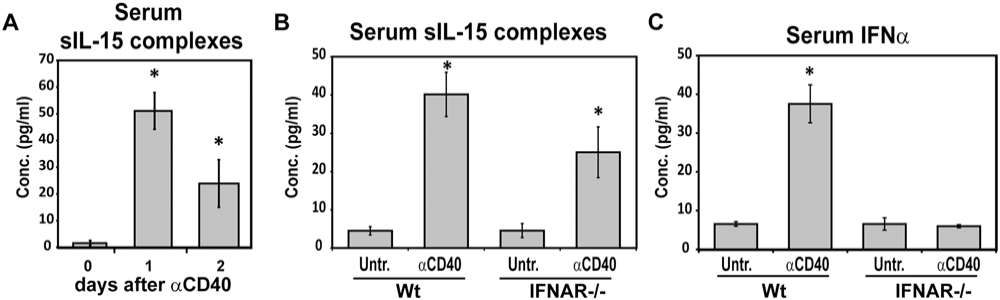
Both IFN and non-IFN pathways induce sIL-15 complexes upon CD40 stimulation. A) Serum was isolated from Wt mice 0, 1, and 2 days after treatment with anti-CD40 mAb (200 μg i.p.). Levels of sIL-15 complexes in serum were measured using ELISA. n = 3–4 mice/group. Similar results were observed in at least 5 experiments. B,C) WT and IFNAR-/- mice were treated with anti-CD40 Ab (50 μg i.p.). Serum was isolated 24 hours later and analyzed for levels of B) sIL-15 complexes and C) IFN-α via ELISA. Graph shows average cytokine levels from 3 experiments. n = 12 mice/group. Error bar represents standard error of the mean. * indicates p<0.05.

Our results suggest that DC stimulation leads to induction of sIL-15 complexes, which under some circumstances is mediated by IFN signaling. CD40 stimulation has been reported to increase surface IL-15 and IL-15Rα on DCs and promote IL-15 responses *in vivo* [23,26] but is not reported as a major inducer of type IFNs. To determine if CD40 stimulation generates sIL-15 complexes, mice were given anti-CD40 Ab (200 μg/mouse) and levels of sIL-15 complexes in the serum were measured. As with VSV infection, anti-CD40 treatment increased sIL-15 complexes to a maximum level one day after treatment (Fig. 4A). Two days after treatment, the levels of sIL-15 complexes were still elevated (Fig. 4A). Similar levels of sIL-15 complexes were induced with lower doses of anti-CD40 Ab (50 μg/mouse) during *in vivo* titration experiments (data not shown). To determine whether sIL-15 complexes could be induced in the absence of IFN signaling, similar experiments were conducted using IFNAR-/- mice. In these mice, treatment with anti-CD40 Ab (50 μg/mouse) significantly increased sIL-15 complexes in the absence of IFNAR but at a reduced level, corresponding to an approximate 40% reduction compared to that induced in Wt mice (Fig. 4B). Because, CD40-mediated increases in sIL-15 complexes were partially reduced in IFNAR-/- mice compared to Wt mice, we determined if IFN-α is produced after CD40 stimulation. Interestingly, IFN-α was present in the serum of CD40 stimulated WT mice but not IFNAR-/- mice (Fig. 4C). These data indicate IFN signaling is only partially required for CD40 mediated increases in sIL-15 complexes. Therefore, CD40 stimulation provides a model where sIL-15 complexes are increased by both IFN and non-IFN pathways.

## Discussion

This study identifies three global inducers of sIL-15 complexes *in vivo*, IFN-α, VSV infection, and CD40 stimulation which can now be included among TLR stimulation and lymphodepletion as events inducing sIL-15 complexes. Type I IFNs and numerous pathogens have been reported to increase IL-15 mRNA and enhance IL-15-dependent responses; however, the regulation of IL-15 has not been well understood because the protein is difficult to detect. The difficulties in detecting IL-15 in biological solutions were due in part because IL-15 is not secreted as a free protein but rather is transported to the cell surface associated with the IL-15Rα, where it is either transpresented to another cell, recycled, or cleaved [6]. Now that we have shown that sIL-15 complexes are produced in response to either IFN-α VSV infection, or CD40 stimulation, there now lies the possibility that increased sIL-15 complexes is the mechanism stimulating IL-15 responsive cells, such as memory CD8 T cells, NK cells, and invariant NKT cells, during these respective circumstances. Since Type I IFNs are produced during numerous inflammatory conditions and multiple types of immune stimulation and we show that IFN-α increases sIL-15 complexes, sIL-15 complexes are likely produced under these diverse circumstances. Moreover, increased production of sIL-15 complexes has also been observed after Influenza infection [36], which could be an indication that production of sIL-15 complexes is a general feature of viral infections. Hence, our study provides evidence that the production of sIL-15 complexes is a much more common event than originally realized and one associated with various forms of inflammation and immune activation.

Our demonstration that IFN-α increases sIL-15 complexes in BMDCs and requires IFNAR signaling identifies Type I IFNs as one direct signaling pathway that induces sIL-15 complexes. Since IFN-α is often elevated in Systemic lupus erythematosus and Psoriasis [37,38], these are conditions that likely exhibit elevated local and/or systemic sIL-15 complexes. Furthermore, we found that continual Type I IFN-signaling *in vivo* can sustain production of sIL-15 complexes, implicating the absence of a negative feedback loop at the level of sIL-15 complex generation. Even though Type I IFNs are induced upon a VSV infection, the production of sIL-15 complexes after VSV infection was unaffected in the absence of Type I IFN signaling showing additional pathways contribute to increased production of sIL-15 complexes. While this result is surprising, it is not unprecedented as *in vivo* injection of LPS has been shown to increase IL-15 transcription in IFNAR-/- mice [20]. The identity of these other signals inducing sIL-15 complexes is currently unclear but may involve signals stimulated by TLRs, additional inflammatory cytokines, and/or cell death. Nonetheless, we find that the generation of sIL-15 complexes induced by TBI and TLR3 stimulation requires Type I IFN signaling showing that in at least these situations, IFN-α is an important pathway regulating sIL-15 complexes.

Whereas Type I IFNs increase the transcription of IL-15 and IL-15Rα and the cell surface expression of IL-15Rα /IL-15 complexes [18–22,24], this current study demonstrates that Type I IFNs also upregulate cleavage of cell surface IL-15Rα /IL-15. Evidence that IFN-α directly regulates cleavage of IL-15 complexes includes our demonstration that ADAM17 is increased in BMDCs by IFN-α and is required for IFN-α-mediated increases in sIL-15 complexes *in vitro*. Interestingly, although increased transcription of IL-15 during a VSV infection requires Type I IFN signaling [18], the abundance of sIL-15 complexes in VSV-infected IFNAR-/- mice provides evidence that the cleavage of IL-15 complexes is not necessarily linked to the transcription of IL-15 and IL-15Rα. Alternatively, increases in IL-15 transcription mediated by IFNAR independent pathways may be occurring earlier than previously investigated. In fact, IL-15 transcription increases in IFNAR-/- mice as early as 4 hrs after LPS treatment [39]. Altogether, these findings show Type I IFNs utilize multiple mechanisms to regulate IL-15 responses, which include regulation of transcription as well as cleavage of cell surface proteins.

Our results showing IFN-α increases the *in vitro* generation of sIL-15 complexes in a manner dependent on ADAM17 is the first account demonstrating Type I IFNs as a regulator of ADAM17 activity. Since ADAM17 mediates the cleavage of numerous other proteins, the regulation of ADAM17 by Type I IFNs is widely relevant. Specifically, ADAM17 mediates the cleavage of TNF-α, sIL-6R, and CD62L, among other proteins [40,41]. As such, our findings reveal a mechanism by which Type I IFNs regulate a group of proinflammatory cytokines and have the capability to alter immune responses during autoimmunity and inflammation-associated conditions.

As shown in this study, VSV infection and CD40 stimulation induce a robust and transient production of sIL-15 complexes. Unfortunately, the role of this burst in sIL-15 complexes early in an infection is unclear as there is presently no *in vivo* model that allows one to distinguish IL-15 responses mediated by sIL-15 complexes from those mediated by transpresentation. However, our data showing that increases in surface IL-15 and sIL-15 complexes are concurrent events suggest both mechanisms are feasible in eliciting IL-15-mediated responses. Nevertheless, it is not unreasonable to speculate that these sIL-15 complexes could enhance immune responses by stimulating NK cells and memory CD8 T cells generated from a prior encounter, as these are two populations very responsive to sIL-15 complexes [11]. These sIL-15 complexes also have the ability to act immediately as they do not require a cell-cell interaction to mediate responses; this is in contrast to transpresentation, which requires immune cells to locate their cellular source for IL-15. Additionally, even though naive CD8 T cells are not as responsive to IL-15 as memory CD8 T cells, it is possible that early exposure to sIL-15 complexes could influence T cell priming. A recent study by Tamzalit F. et al [42] provides evidence that cleavage of IL-15Rα /IL-15 complexes from the surface is important for efficient translocation and internalization of IL-15 complexes to opposing cells during transpresentation [42]. If this is the case, then the production of sIL-15 complexes we observe may be a biomarker of concurrently enhanced IL-15 transpresentation. But more importantly, this scenario doesn’t discount those circulating sIL-15 complexes, which have agonistic activity, could be acting on IL-15-responsive cells.

The induction of lymphopenia is well known to enhance lymphocyte responses to homeostatic cytokines, such as IL-15 and IL-7 [43–45]; however, the mechanism responsible for enhanced IL-15 responses has been unclear. Hence, the demonstration that sIL-15 complexes are induced by TBI or chemotherapy [10] gives credence to the idea that elevated sIL-15 complexes are contributing to enhanced lymphocyte responses during lymphopenic conditions. Increasing lymphocyte responses during lymphopenia is very clinically relevant as lymphodepletion is widely used as an integral component of immunotherapy in the treatment of cancers. While studies have clearly demonstrated that IL-15 is critical for enhanced anti-tumor responses in mouse models of adoptive transfer [46], as mentioned before, there are currently no models to discern whether these IL-15-mediated responses are due to transpresented IL-15 or sIL-15 complexes. Therefore, the potential mechanisms mediating IL-15 responses during lymphopenic conditions should now include those elicited by transpresented IL-15, sIL-15 complexes, or the combination of both.

In summary, sIL-15 complexes are increased by IFN-α (a common pro-inflammatory factor), VSV infection, and CD40 stimulation. These findings along with previous studies showing TLR stimulation and lymphodepletion induce sIL-15 complexes leads us to conclude that increases in sIL-15 complexes is an event associated with inflammation and immune activation. Moreover, the wide-ranging nature of these stimuli suggests that induction of sIL-15 complexes during immune stimulation is a frequent event. These observations provide an unrealized mechanism for enhanced IL-15 responses observed in response to Type I IFNs and other inflammatory settings.
